# Adapting Local Features for Face Detection in Thermal Image

**DOI:** 10.3390/s17122741

**Published:** 2017-11-27

**Authors:** Chao Ma, Ngo Thanh Trung, Hideaki Uchiyama, Hajime Nagahara, Atsushi Shimada, Rin-ichiro Taniguchi

**Affiliations:** 1 Graduate School of Information Science and Electrical Engineering, Kyushu University, 744, Motooka, Nishi-ku, Fukuoka 819-0395, Japan; uchiyama@limu.ait.kyushu-u.ac.jp (H.U.); atsushi@limu.ait.kyushu-u.ac.jp (A.S.); rin@kyudai.jp (R.T.); 2 The Institute of Scientific and Industrial Research, Osaka University, 8-1 Mihogaoka, Ibaraki, Osaka 567-0047, Japan; trung@am.sanken.osaka-u.ac.jp; 3 Institute for Datability Science, Osaka University, 2-8, Yamadaoka, Suita, Osaka 565-0871, Japan; nagahara@ids.osaka-u.ac.jp

**Keywords:** thermal image, face detection, mixed features, haar-like, histogram of oriented gradient, local binary pattern, local ternary pattern, AdaBoost

## Abstract

A thermal camera captures the temperature distribution of a scene as a thermal image. In thermal images, facial appearances of different people under different lighting conditions are similar. This is because facial temperature distribution is generally constant and not affected by lighting condition. This similarity in face appearances is advantageous for face detection. To detect faces in thermal images, cascade classifiers with Haar-like features are generally used. However, there are few studies exploring the local features for face detection in thermal images. In this paper, we introduce two approaches relying on local features for face detection in thermal images. First, we create new feature types by extending Multi-Block LBP. We consider a margin around the reference and the generally constant distribution of facial temperature. In this way, we make the features more robust to image noise and more effective for face detection in thermal images. Second, we propose an AdaBoost-based training method to get cascade classifiers with multiple types of local features. These feature types have different advantages. In this way we enhance the description power of local features. We did a hold-out validation experiment and a field experiment. In the hold-out validation experiment, we captured a dataset from 20 participants, comprising 14 males and 6 females. For each participant, we captured 420 images with 10 variations in camera distance, 21 poses, and 2 appearances (participant with/without glasses). We compared the performance of cascade classifiers trained by different sets of the features. The experiment results showed that the proposed approaches effectively improve the performance of face detection in thermal images. In the field experiment, we compared the face detection performance in realistic scenes using thermal and RGB images, and gave discussion based on the results.

## 1. Introduction

Face detection is fundamental in computer vision. Most of the existing works on face detection are based on visible images, because RGB color cameras are easy to obtain. Face detection in visible images has been around for decades with various problems tackled. However, there are still some problems which have not been completely solved. We can see three main problems. (1) Facial appearances in visible images are liable to change by lighting condition [1]. For example, when the light source is on the right side of a face, this side of facial region appears brighter than that of left side in a visible image, and vice versa. It is not so easy for a face detection algorithm to find facial regions with different brightness distribution simultaneously. (2) Facial appearances in visible images sometimes become irregular by make-up pigments [2]. For example, actors/actresses in dramas or fashion shows sometimes wear strange make-up, which makes the facial appearances looked totally unhuman. In this situation, detecting faces is basically impossible using visible images. (3) It is hard to discriminate printed faces from real ones in visible images by almost all face detection algorithms [3]. The reason is printed faces are the same as real faces in visible images. To completely solve all the above problems is quite difficult, since these problems are inevitable when using visible images. We need to find a new camera type which can provide more reliable information under those adverse conditions.

There are a variety of digital cameras in these days with different properties besides RGB color cameras. A thermal camera is one of them and works in thermal IR band (wavelength between 8–14 μm). The light in this band is called thermal IR radiation. The strength of thermal IR radiation from an object is associated with its temperature [4]. In a thermal camera, a germanium lens images the thermal IR radiation from the scenes to a thermal sensor (also called infrared focal plane array). In the thermal image sensor, each pixel is a small thermal IR radiation detector made of thermal IR radiation sensitive material [4]. By this way, thermal cameras can obtain the thermal distribution of the scenes as thermal images. They are the potential alternatives for face detection applications.

We can see there are three advantages to detect faces using thermal cameras. (1) The temperature distribution of human faces is generally constant and higher than backgrounds [5]. This advantage solves 2 problems: (i) Printed faces can be discriminated from real ones in thermal images. Printed faces have different temperature distribution from real ones. As a result, they are different with real ones in thermal images; (ii) Faces with strange make-up pigments are easier to detect in thermal images than those in visible images. Make-up pigments do not affect the facial temperature distribution. As a result, a face with or without make-up pigments appears the same in thermal images. (2) Thermal cameras are only sensitive to the radiation from thermal IR band, while not sensitive to visible light. This means no matter how we change the ambient lighting condition (e.g., strength, color or direction of light), the facial appearances keep the same in thermal images. This advantage solves the problem that facial appearances dramatically change under different lighting conditions in visible images. (3) Using thermal cameras for face detection can achieve better privacy protection. It is harder to recognize the identities of people in thermal images [6]. The reason is in thermal images, due to the similar facial temperature distribution, human faces appear much more similar than those in visible images.

There are very few works on face detection using thermal images compared with those using visible images. In the existing works, the most commonly used approach is cascade classifiers with Haar-like features [7]. Other than Haar-like features, there are feature types proposed, such as Multi-Block LBP [8] and HOG [9]. However, the existing feature types are designed based on visible images, since RGB color cameras are intensively used. There is no feature type especially designed for face detection in thermal images. This means no feature type considers the properties of facial regions in thermal images, such as the generally constant temperature distribution.

Moreover, we can see different feature types have different advantages. For example, Haar-like is adept at describing contrast of local neighbors [10], Multi-Block LBP is adept at describing local textures [8], HOG is adept at describing local edges [11]. We can group these feature types into two categories with respect to AdaBoost algorithm: (1) number-type features, such as Haar-like or HOG (HOG feature was originally proposed as histogram representation. It can also be used as number representation with respect to AdaBoost algorithm, where each bin of a HOG histogram is considered as a HOG feature (see the second paragraph of 2.1 for details). In this paper, we will use the name of HOG as number representation when talking about AdaBoost algorithm); (2) category-type features, such as Multi-Block LBP [12]. The response of a number-type feature is a real number, while that of a category-type feature is a high-dimensional vector [12]. To enhance the description power of features, Xia et al. [13] proposed a method to use multiple feature types in one cascade classifier. However, their method can only employ two number-type features (Haar-like and HOG). To improve description power further, we need a method to train a cascade classifier containing both number-type and category-type features. Furthermore, as far as we surveyed, there is no work on face detection in thermal images using a cascade classifier containing multiple feature types.

In our research, we detect faces in thermal images by using cascade classifiers. Our work is based on Adaboost algorithm with local features. We have two contributions:

- We create new feature types by considering the properties of facial regions in thermal images. We realize our new feature types by extending Multi-Block LBP. We consider 2 aspects: (1) A margin around the reference; (2) The generally constant distribution of facial temperature. In this way we make the features more robust to image noise and more effective for face detection in thermal images.
- We propose an AdaBoost-based training method to build cascade classifiers containing different feature types with different advantages. Our algorithm can build cascade classifiers containing number-type and/or category-type features. In this way we can obtain an improved description power.

For the first contribution, we realize it by extending Multi-Block LBP in two steps. In step one, we improve robustness of Multi-Block LBP to thermal camera noise by considering a margin around the central reference. The idea is similar to the extension from Local Binary Pattern (LBP) [14] to Local Ternary Pattern (LTP) [15]. The difference is our feature type is for AdaBoost algorithm. We call our new feature type Multi-Block LTP. In step two, we adapt both Multi-Block LBP and Multi-Block LTP more effective for face detection in thermal images. We change the reference to an absolute temperature according to the generally constant distribution of facial temperature. The reference temperature is optimized by Adaboost algorithm for each selected feature. We call them Absolute Multi-Block LBP (AMB-LBP) and Absolute Multi-Block LTP (AMB-LTP), respectively. As far as we know, our AMB-LBP and AMB-LTP are the first two local feature types that consider the temperature of thermal images. In summary, we present three extensions of the original Multi-Block LBP. In this way, we get a set of extended feature types including the original one: {Multi-Block LBP, Multi-Block LTP, AMB-LBP, AMB-LTP}. For the second contribution, it is the extension of our conference paper [16]. In that paper we only described the idea to build a cascade classifier containing multiple existing feature types. In this paper, we build cascade classifiers also containing our new feature types, and add more experiment results and discussion.

We structure this paper as follows. Section 2 describes the related works. Section 3 describes our first contribution: our new feature types by extending Multi-Block LBP. Section 4 describes our second contribution: the AdaBoost-based training method to build a cascade classifier with multiple feature types. Section 5 describes the hold-out validation experiment. In the experiment, we captured the dataset and compared the cascade classifiers trained by using regular feature types, our new feature types, and multiple feature types. Section 6 describes the field experiment. In the experiment, we built up several natural scenes and tested face detection performance of thermal images as well as that of RGB images. Section 7 concludes our works and gives future work directions.

## 2. Related Work

### 2.1. Local Features

Haar-like features were first proposed by Viola and Jones [17] in 2001 for face detection using visible images. The response of one Haar-like feature was calculated by subtracting sums of pixel values in neighboring rectangle regions. All the Haar-like features formed a feature pool. AdaBoost algorithm was employed to repeatedly select a feature from the feature pool for a weak classifier. Several weak classifiers were combined into a strong classifier. After several strong classifiers were built, they were chained together into a cascade classifier. Their cascade classifier was the first real-time high-performance method for face detection using visible images [1]. Later, Reese et al. [7] proved that it is also feasible to employ a Haar-like-based cascade classifier for face detection using thermal images. The limitation of the Haar-like features is that they are too simple [8]. As a consequence, the obtained cascade classifier contains too many weak classifiers and leads to high computation.

Histogram of Oriented Gradient (HOG) was first proposed by Dalal and Triggs [9] in 2005 for human body detection using visible images. Its response was calculated as a histogram using all the pixels in image patches. The calculation of the response histogram contained two steps. First, image gradients were calculated pixel by pixel in image patches. Second, response histogram formed according to the gradient orientations. HOG feature also can be employed by AdaBoost algorithm. For example, Jia and Zhang [18] proposed a HOG-based cascade classifier. To fit the AdaBoost framework, they used a lower level form of HOG. Their lower lever HOG was defined by the value of one orientation bin in the original HOG response histogram. In our work, we also adopt that lower level form since we also use AdaBoost algorithm. The limitation of HOG is that it works efficiently only for objects with clear contours. The reason is that HOG is calculated by considering image gradients, which indicate the edges in images well [19].

Local Binary Pattern (LBP) was first proposed by Ojala et al. [14] in 1996 for texture analysis using visible images. Its response was calculated as a histogram. The calculation of its response histogram contained two steps. First, each pixel value in image patches was compared as reference with those of the surrounding ones. If the surrounding pixel was larger than the center one, the place of the surrounding pixel was labeled as 1. Otherwise, it was labeled as 0. The resulted vectors of 0 and 1 calculated by all the pixels were the response patterns. Second, the response histogram was formed according to different response patterns. The limitation of LBP feature is its sensitivity to noise for the uniform image regions [15]. The reason is that in uniform image regions, pixel intensities are quite similar. When tiny image noise pollute a pixel, the comparison result of one pixel with its surrounding ones is possibly changed. This causes an incorrect LBP response pattern. Too many incorrect LBP response patterns will finally lead to an incorrect LBP response histogram. Unfortunately, lots of uniform regions exist in facial regions [15], which causes the unrobustness for facial analysis using the original LBP.

To improve the robustness of LBP, there are two main approaches. First approach is introducing a margin around the central reference pixel value when calculating the response patterns. By this way, the central reference becomes a range rather than a fixed value. When tiny image noise exists, this margin can keep the response pattern same. A typical feature type adopts this approach is Local Ternary Pattern (LTP) [15]. Second approach is comparing average pixel values in blocks with multiple pixels. In this way, the interference of image noise is canceled by averaging. A typical feature type adopts this approach is Multi-scale Block LBP [20]. Interestingly, there are also feature types considering both of these approaches. Multi-scale Block LTP [21] is one of them.

The LBP, LTP, Multi-scale Block LBP and Multi-scale Block LTP are calculated in all possible position inside image patches for response histograms. This calculation is quite time consuming for real-time face detection in video. Furthermore, these feature types cannot be employed directly by cascade classifiers, because they use histogram representations. To achieve a faster face detection speed, Multi-Block LBP features were proposed by Zhang et al. [8] for AdaBoost algorithm. Multi-Block LBP also compared the average pixel value of the central multi-pixel block with those of the surrounding ones as a response pattern. However, the response of Multi-Block LBP was not represented by a histogram. The response patterns were directly used for classification in weak classifiers of cascade classifiers.

In the original papers introducing Multi-scale Block LBP [20] and Multi-Block LBP [8], both of them used the abbreviation of MB-LBP. The paper introducing Multi-scale Block LTP [21] used the abbreviation of MB-LTP. However, as we described, multi-block features and multi-scale block features are different. To discriminate them, in this paper, we will use the full name in the places where ambiguity may arise. Furthermore, the use of MB-LBP and MB-LTP in this paper is for Multi-Block LBP by Zhang et al. [8] and our new feature type, Multi-Block LTP, respectively.

From all the feature types above we can see two aspects: (1) The feature types which can be employed by cascade classifiers fall into two groups: (i) Number-type features, such as Haar-like or HOG; (ii) Category-type features, such as Multi-Block LBP [12]. The response of a number-type feature is a real number, while that of a category-type feature is a high-dimensional vector [12]. Our proposed cascade classifiers contain both number-type and category-type features. (2) The category-type features are relatively complex, because the dimension of their responses is higher. This complexity gives a room to further improve them. Our new feature types are based on the extension of Multi-Block LBP.

### 2.2. Fusion of Features

Different feature types have different description power. To enhance the feature description power, multiple feature types are used together rather than just single type. There are three main approaches to combine multiple feature types in object detection applications: concatenation, co-occurrence, and mixed-feature pool. In our survey, all approaches have only been used in visible images.

Concatenation is realized by concatenating several individual feature histograms of different feature types to a longer one. This requires the used feature types have histogram representations. Wang et al. [11] proposed a method to concatenate histogram of LBP and that of HOG to a HOG-LBP histogram. Jiang and Ma [22] created color and bar-shaped feature histograms, and concatenated them with HOG histogram to obtain a feature type called HOG III. Both of these two concatenated feature types were used for human body detection. They achieved an improved performance compared with those only using one feature type.

Co-occurrence is realized by using more than one features simultaneously for one weak classifier in cascade classifiers. Mita et al. [23] proposed a joint Haar-like features for face detection. Their joint features were implemented using two or three co-occurring Haar-like features. They showed that their joint features were better than the single Haar-like feature.

Mixed feature pool is realized by mixing two or more feature types in one feature pool for the feature selection by AdaBoost algorithm. As a result, one strong classifier in cascade classifiers may be built by weak classifiers with different feature types. The difference from the co-occurrence approach is that one weak classifier contains only one feature. Xia et al. [13] proposed a mixed feature pool for an object-tracking application using visible images. They mixed two number-type features, Haar-like and HOG, in their mixed feature pool. We expect that the mixed feature pool can also be applied to thermal images. Furthermore, we see employing only number-type features is limiting because category-type features such as Multi-Block LBP are not considered. In our work, we create a mixed feature pool containing both number-type and category-type features. We propose an AdaBoost-based training method to build cascade classifiers with number-type and/or category-type features. Our training method differ from that of Xia et al. [13]. Ours can select features from feature pools containing both number-type and category-type features. In contrast, theirs can only select features from feature pool with number-type features (Haar-like and HOG).

## 3. Extension of Multi-Block LBP Feature

In this section, we will present our new feature types. In Section 3.1, we extend Multi-Block LBP to Multi-Block LTP by considering a margin for better robustness to thermal camera noise. In Section 3.2, we extend the two feature types, Multi-Block LBP and Multi-Block LTP described in Section 3.1 by considering the generally constant distribution of facial temperature to improve the performance for face detection in thermal images. We discuss all the feature types in the setting of thermal images. We use pixel temperature instead of pixel value, since the pixel value represents the temperature.

### 3.1. Multi-Block Local Ternary Patterns

Multi-Block LBP [8] encodes the texture around a multi-pixel block. It is defined by comparing the average pixel temperature of the central block as reference with those of its 8 surrounding blocks, the feature response on image patch $x_{i}$ is encoded by an integer:

(1) $$P_{Multi-Block\;LBP}\left(x_{i}\right)=\Sigma_{q=0}^{7}2^{q}S_{Multi-Block\;LBP}\left(g_{q}-g_{c}\right),$$

where $g_{c}$ and $g_{q}$ represent the average pixel temperatures of the center block and that of one of the 8 blocks surround it with index *q*. $S_{Multi-Block\;LBP}$ is the labeling function defined by:

(2) $$S_{Multi-Block\;LBP}\left(u\right)=\left\{\begin{array}{cc} 1 & u\geq 0\\ 0 & u<0. \end{array}\right.$$

**Figure 1:**
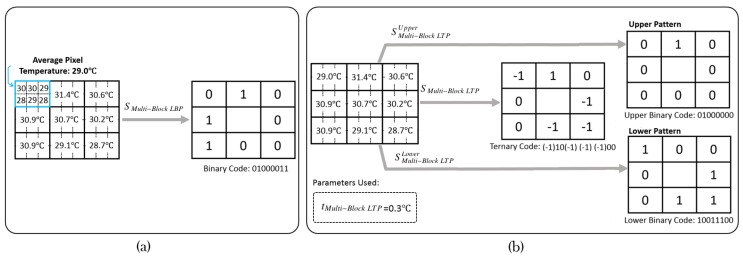
Examples demonstrating the calculation of Multi-Block LBP and Multi-Block LTP. Each rectangular region has 2 × 3 pixels. (**a**) The example of calculation of Multi-Block LBP. (**b**) The example of calculation of Multi-Block LTP with $t_{Multi-Block\;LTP}=0.3{\;}^{\circ }$C.

We can choose any order in determining the index *q* of the surrounding blocks when encoding. Zhang et al. [8] encoded clockwise from the top left block, which we will follow. Figure 1a illustrates the encoding process of Multi-Block LBP with an example.

To improve the robustness of Multi-Block LBP to thermal camera noise, we extend it by considering a margin around the reference. We use three values to label the comparison result. The idea is similar to the extension from LBP to LTP by Tan and Triggs [15], or the extension from Multi-scale Block LBP to Multi-scale Block LTP by Jia et al. [21]. The difference is that our extended feature type is for AdaBoost algorithm. We call our new feature Multi-Block LTP.

We use $S_{Multi-Block\;LTP}\left(\left(g_{q}-g_{c}\right),t_{Multi-Block\;LTP}\right)$ to label the 8 surrounding blocks, the labeling function is defined as:

(3) $$S_{Multi-Block\;LTP}\left(u,t\right)=\left\{\begin{array}{cc} 1 & u\geq t\\ 0 & -t<u<t\\ -1 & u\leq -t. \end{array}\right.$$

$g_{c}$ and $g_{q}$ also represent the average pixel temperature of the center block and that of one of the 8 surrounding blocks with index *q*. The margin $t_{Multi-Block\;LTP}$ $\left(t_{Multi-Block\;LTP}\geq 0\right)$ is optimized for each feature as a feature parameter in the training process, similar to the position or size of the feature.

In implementation, to keep the dimension of feature lookup table low for efficiency [15], we convert one ternary pattern feature to an upper and a lower binary pattern features similar to the method by Tan and Triggs [15]. The upper and lower binary pattern features of Multi-Block LTP on image patch $x_{i}$ are encoded by integers $P_{Multi-Block\;LTP}^{Upper}\left(x_{i}\right)$ and $P_{Multi-Block\;LTP}^{Lower}\left(x_{i}\right)$ respectively,

(4) $$P_{Multi-Block\;LTP}^{Upper}\left(x_{i}\right)=\Sigma_{q=0}^{7}2^{q}S_{Multi-Block\;LTP}^{Upper}\left(\left(g_{q}-g_{c}\right),t_{Multi-Block\;LTP}\right),$$

(5) $$P_{Multi-Block\;LTP}^{Lower}\left(x_{i}\right)=\Sigma_{q=0}^{7}2^{q}S_{Multi-Block\;LTP}^{Lower}\left(\left(g_{q}-g_{c}\right),t_{Multi-Block\;LTP}\right),$$

where

(6) $$S_{Multi-Block\;LTP}^{Upper}\left(u,t\right)=\left\{\begin{array}{cc} 1 & u\geq t\\ 0 & otherwise, \end{array}\right.$$

and

(7) $$S_{Multi-Block\;LTP}^{Lower}\left(u,t\right)=\left\{\begin{array}{cc} 1 & u\leq -t\\ 0 & otherwise. \end{array}\right.$$

In this way, one Multi-Block LTP feature can be deemed as binary pattern feature pair. For training by AdaBoost, employing Multi-Block LTP means selecting in all the upper and lower binary pattern features independently. For detecting, only the selected upper or lower pattern features are used. We demonstrate the example of Multi-Block LTP encoding process in Figure 1b. In our implementation, we also encode the Multi-Block LTP and its upper and lower patterns clockwise from top left block.

### 3.2. Absolute Multi-Block LBP and Absolute Multi-Block LTP

**Figure 2:**
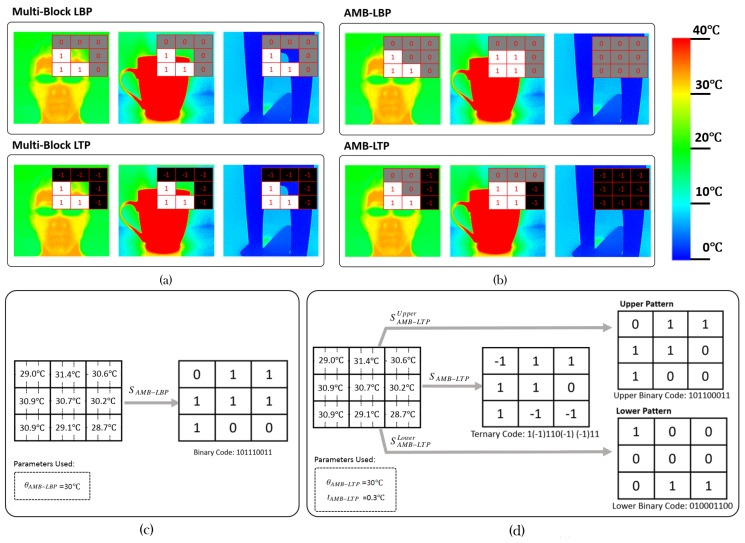
(**a**) The responses of Multi-Block LBP and Multi-Block LTP with the same size and location, while $t_{Multi-Block\;LTP}=0.3$ ${}^{\circ }$C . The three images contain a male face, a hot cup and a book stand. The white, gray and black blocks of the feature represent the codes of 1, 0 and −1. In this situation, Multi-Block LBP and Multi-Block LTP fail to discriminate the face, hot cup, and book stand. (**b**) The responses of AMB-LBP and AMB-LTP features with the same size and location as those in (**a**). The example use the absolute temperature 30 ${}^{\circ }$C as reference for both AMB-LBP and AMB-LTP, and $t_{AMB-LTP}$ is set to 0.3 ${}^{\circ }$C. In this situation, AMB-LBP and AMB-LTP can clearly discriminate the three objects. (**c**,**d**) are the examples of calculation of AMB-LBP and AMB-LTP, respectively.

Multi-Block LBP and Multi-Block LTP compare the average pixel temperature of the central block with those of surrounding ones. This relative comparison is not always efficient for thermal images. We can find lots of non-facical patches with the same responses as those of the facical patches for a specific feature. Figure 2a illustrates the phenomenon by showing the responses of the same positioned Multi-Block LBP and Multi-Block LTP features in three scenes containing different objects. We can clearly see that they have the same response patterns, which means the used features fail to discriminate face from other objects.

To improve the effectiveness of Multi-Block LBP and Multi-Block LTP for face detection in thermal images, we notice that facial temperature distribution is generally constant. This suggests a way to improve the discriminative abilities of Multi-Block LBP and Multi-Block LTP. We change the reference to an absolute temperature θ. This temperature is optimized by Adaboost algorithm for each selected feature. We call the new feature types Absolute Multi-Block LBP (AMB-LBP) and Absolute Multi-Block LTP (AMB-LTP). In Multi-Block LBP or Multi-Block LTP, we encode the 8 surrounding blocks. However, since the absolute reference temperature does not belong to any block for AMB-LBP or AMB-LTP, we encode all the 9 blocks same as the approach by Jin et al. [24]. The one more bit is encoded at the very beginning before the 8-bit clockwise coding sequence from the top left block.

We encode an AMB-LBP feature by an integer:

(8) $$P_{AMB-LBP}\left(x_{i}\right)=\Sigma_{a=0}^{8}2^{a}S_{AMB-LBP}\left(g_{a},\theta_{AMB-LBP}\right),$$

where $g_{a}$ represents the average pixel temperature of one of the total nine blocks, its index is *a*. The labeling function $S_{AMB-LBP}$ is defined as:

(9) $$S_{AMB-LBP}\left(\lambda ,\theta \right)=\left\{\begin{array}{cc} 1 & \left(\lambda -\theta \right)\geq 0\\ 0 & \left(\lambda -\theta \right)<0, \end{array}\right.$$

where $\theta_{AMB-LBP}$ is the absolute reference temperature, which is optimized in the training process for each feature as one feature parameter.

For AMB-LTP, we use $S_{AMB-LTP}\left(g_{a},\theta_{AMB-LTP},t_{AMB-LTP}\right)$ to label all the blocks, each block has the index *a*. The labeling function is defined as:

(10) $$S_{AMB-LTP}\left(\lambda ,\theta ,t\right)=\left\{\begin{array}{cc} 1 & \left(\lambda -\theta \right)\geq t\\ 0 & -t<\left(\lambda -\theta \right)<t\\ -1 & \left(\lambda -\theta \right)\leq -t. \end{array}\right.$$

Similar to Multi-Block LTP, we convert the ternary pattern into an upper binary pattern $P_{AMB-LTP}^{Upper}\left(x_{i}\right)$ and a lower binary pattern $P_{AMB-LTP}^{Lower}\left(x_{i}\right)$,

(11) $$P_{AMB-LTP}^{Upper}\left(x_{i}\right)=\Sigma_{a=0}^{8}2^{a}S_{AMB-LTP}^{Upper}\left(g_{a},\theta_{AMB-LTP},t_{AMB-LTP}\right),$$

(12) $$P_{AMB-LTP}^{Lower}\left(x_{i}\right)=\Sigma_{a=0}^{8}2^{a}S_{AMB-LTP}^{Lower}\left(g_{a},\theta_{AMB-LTP},t_{AMB-LTP}\right),$$

where

(13) $$S_{AMB-LTP}^{Upper}\left(\lambda ,\theta ,t\right)=\left\{\begin{array}{cc} 1 & \left(\lambda -\theta \right)\geq t\\ 0 & otherwise, \end{array}\right.$$

and

(14) $$S_{AMB-LTP}^{Lower}\left(\lambda ,\theta ,t\right)=\left\{\begin{array}{cc} 1 & \left(\lambda -\theta \right)\leq -t\\ 0 & otherwise, \end{array}\right.$$

where $g_{a}$ represents the average pixel temperature of one of the nine blocks with index *a*, The margin $t_{AMB-LTP}$ and absolute reference temperature $\theta_{AMB-LTP}$ are optimized in the training process for each feature as feature parameters. The upper and lower binary pattern features of AMB-LTP are used in the same way for training and detecting as that of Multi-Block LTP.

Figure 2c,d illustrate the encoding processes of AMB-LBP and AMB-LTP by examples. Because AMB-LBP and AMB-LTP consider the generally constant distribution of facial temperature, we can see from Figure 2b that both of them can discriminate faces from other two objects in the example.

Similar to the advantage of robustness to thermal camera noise of Multi-Block LTP over Multi-Block LBP, we can expect an improved robustness to thermal camera noise from AMB-LTP over AMB-LBP. On the other hand, because the calculation process of AMB-LBP is simpler than that of AMB-LTP, which means a faster calculation speed. It is meaningful for embedded system application if the performance of AMB-LBP is not too far from that of AMB-LTP.

## 4. Learning Mixed Features

### 4.1. Overview

We propose an AdaBoost-based training algorithm to train a cascade classifier. The input for our algorithm includes a sample pool, and a feature pool. The output is a cascade classifier with a chain of strong classifiers.

**Figure 3:**
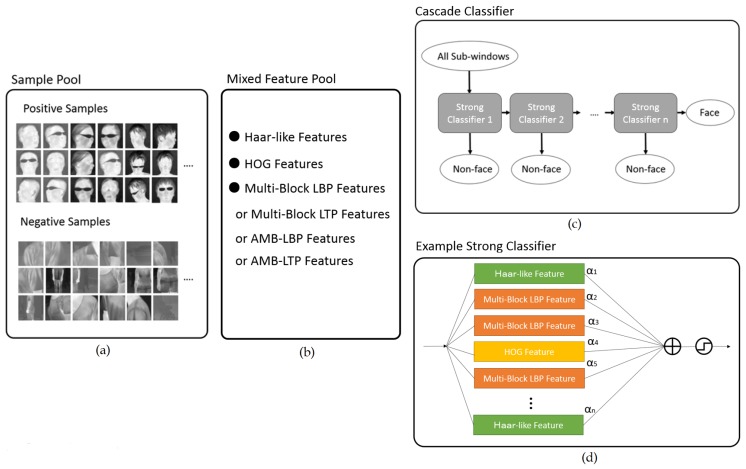
Concept of the mixed feature training. (**a**) Sample pool. (**b**) Mixed feature pool containing different feature types. (**c**) Resulting cascade classifier. (**d**) Example of a strong classifier trained by our algorithm. Each feature is the best from the mixed feature pool {Haar-like Features, HOG Features, Multi-Block LBP Features} in its selection iteration. In this way, a strong classifier may contain features of different types.

With respect to the input, we use facial and non-facial patches in thermal images as positive and negative samples, respectively. Figure 3a illustrates the sample pool that we use. We expect to take advantage of different description power of different feature types. We mix Haar-like, HOG, and one feature type in the set {Multi-Block LBP, Multi-Block LTP, AMB-LBP, AMB-LTP} to our feature pool. The reason we use only one feature type in the set is that all feature types in it have similar properties and advantages, using more than one is redundant. We build up a mixed feature pool as illustrated in Figure 3b.

With respect to the output, we obtain a cascade classifier from our algorithm given the input above. Figure 3c illustrates the resulting cascade classifier, which is composed of a chain of strong classifiers. In building one strong classifier, our algorithm repeatedly selects the best feature from the mixed feature pool. Using the selected feature, a weak classifier is built for a strong classifier under construction. The building of a strong classifier is finished until it meets the predefined requirements in minimum detection rate (DR) and maximum false alarm rate (FAR). In this way, a strong classifier may contain multiple feature types. Figure 3d illustrates an example strong classifier.

Our training algorithm is similar to that by Viola and Jones [17]. The main difference is that our feature pool contains multiple feature types, while their feature pool contains only Haar-like features. As a result, the building of one strong classifier differs from theirs. In our approach, we need to compare the performance of features from same type as well as different types, and select the best one. Meanwhile, the approach of Viola and Jones only compare Haar-like features and select the best one. The following section will describe how we build a strong classifier.

### 4.2. Building One Strong Classifier

A strong classifier consists of many voters, with each voter including a weak classifier and its weight. This weight is determined in the training process. A weak classifier includes prediction function for one feature type and a feature of that type. When a weak classifier classifies a sample, first it calculates the feature response, then it applies the prediction function to the response for prediction. There are two types of weak classifiers associated with the two feature types. For a weak classifier containing a number-type feature *m*, the prediction function $h_{m}$ on sample $x_{i}$ is

(15) $$h_{m}\left(x_{i}\right)=\left\{\begin{array}{cc} 0 & \mathrm{if}\;d_{m}R_{m}\left(x_{i}\right)<d_{m}T_{m}\\ 1 & \mathrm{otherwise}, \end{array}\right.$$

where $R_{m}\left(x_{i}\right)$ represents the real-number response of the feature *m* on the sample $x_{i}$, and $T_{m}$ is the threshold of the feature *m*. This threshold is optimized in the training process. $d_{m}\in \left\{-1,1\right\}$ is a directional factor indicating the direction of the inequality sign. Its value is also optimized in the training process [17]. For a weak classifier containing a category-type feature *n*, the prediction function $h_{n}$ on sample $x_{i}$ is

(16) $$h_{n}\left(x_{i}\right)=LUT_{n}\left(P_{n}\left(x_{i}\right)\right),$$

where $P_{n}\left(x_{i}\right)$ represents the response vector of the feature *n* on the sample $x_{i}$, $LUT_{n}$ represents the lookup table operator for feature *n*, $LUT_{n}\left(P_{n}\left(x_{i}\right)\right)\in \left\{0,1\right\}$. This lookup table is optimized in the training process [8]. In summary, constructing a weak classifier needs two works. (1) Determining $\left(d_{m},T_{m}\right)$ or $LUT_{n}$ if the feature is number-type or category-type feature, respectively. (2) After $\left(d_{m},T_{m}\right)$ and $LUT_{n}$ are determined for all the features in the mixed feature pool, selecting the best feature *v* from the mixed feature pool.

To determine $\left(d_{m},T_{m}\right)$ for a number-type features *m*, the method in [17] is used. To be specific, $\left(d_{m},T_{m}\right)$ is optimized by:

(17) $$\left(d_{m},T_{m}\right)=\underset{d,T}{\arg\min}\sum_{x_{i}\in \Omega }w_{k,i}\left|\varepsilon \left[d\left(R_{m}\left(x_{i}\right)-T\right)\right]-y_{i}\right|,$$

where Ω represents all the training samples, $w_{k,i}$ is the weight of sample $x_{i}$ at iteration *k*. $y_{i}=0$ for negative samples and $y_{i}=1$ for positive samples. ε is defined as:

(18) $$\varepsilon \left(\lambda \right)=\left\{\begin{array}{cc} 1 & \lambda \geq 0\\ 0 & otherwise. \end{array}\right.$$

To determine $LUT_{n}$ for category-type features, the method in [8] is used. To be specific, the $LUT_{n}$ can be expressed by:

(19) $$LUT_{n}\left(P_{n}\left(x_{i}\right)\right)=\left\{\begin{array}{cc} a_{0} & I\left(P_{n}\left(x_{i}\right)\right)=0\\ & \ldots \\ a_{q} & I\left(P_{n}\left(x_{i}\right)\right)=q\\ & \ldots \\ a_{d} & I\left(P_{n}\left(x_{i}\right)\right)=d. \end{array}\right.$$

where $I\left(P_{n}\left(x_{i}\right)\right)$ is the index number of the response vector of $P_{n}\left(x_{i}\right)$ in all kinds of response vectors. $a_{d}\in \left\{0,1\right\}$ is the binary prediction result. For Multi-Block LBP or Multi-Block LTP, d=255, while for AMB-LBP or AMB-LTP, d=511. $a_{q}$ is determined as:

(20) $$a_{q}=\varepsilon \left(\frac{\sum_{x_{i}\in \Omega }w_{k,i}y_{i}\delta \left(I\left(P_{n}\left(x_{i}\right)-q\right)\right)}{\sum_{x_{i}\in \Omega }w_{k,i}\delta \left(I\left(P_{n}\left(x_{i}\right)-q\right)\right)}-\frac{1}{2}\right),$$

where

(21) $$\delta \left(\lambda \right)=\left\{\begin{array}{cc} 1 & \lambda =0\\ 0 & otherwise. \end{array}\right.$$

After the $\left(d_{m},T_{m}\right)$ and $LUT_{n}$ are decided for all number-type and category-type features in the mixed feature pool, the best feature *v* is selected from it. The rule for selection is by minimizing the error:

(22) $$e_{l}=\sum_{i=1}^{r}w_{k,i}\left|h_{l}\left(x_{i}\right)-y_{i}\right|,$$

where $h_{l}$ is the prediction function of feature *l* in the mixed feature pool. In minimizing $e_{l}$ in (22), the prediction functions (15) and (16) are used for number-type and category-type features, respectively. With the minimization of $e_{l}$, the algorithm finds the best feature *v* in the mixed feature pool as:

(23) $$v=\underset{l}{\arg\min}e_{l}.$$

The whole algorithm for building a strong classifier is as follows:

- **Input:**
  1. *r* training samples $\left\{\left(x_{i},y_{i}\right)\right\}$ where $y_{i}=0$ for the *s* negative samples and $y_{i}=1$ for the *t* positive samples.
  2. Mixed feature pool: $MFP=\left\{l\right\}$.
  3. User defined training parameter: minimum detection rate (DR), and maximum false alarm rate (FAR) for one strong classifier.
- **Output:**
  1. Feature set $F=\left\{m\right\}\cup \left\{n\right\}$, its associated voter set $U=\left\{(m,d_{m},T_{m},\alpha_{m})\right\}\cup \left\{(n,LUT_{n},\alpha_{n})\right\}$, where $\left\{(m,d_{m},T_{m},\alpha_{m})\right\}$ for number-type features, and $\left\{(n,LUT_{n},\alpha_{n})\right\}$ for category-type features. $\alpha_{m}$ and $\alpha_{n}$ represent the weights of the weak classifiers with features *m* and *n*, respectively.
  2. A strong classifier built from *U*, with a trained threshold *T*, its prediction function *H* on sample $x_{i}$ is:
    (24) $$H\left(x_{i};U\right)=\left\{\begin{array}{cc} 1 & \sum_{f\in F}\alpha_{f}h_{f}\left(x_{i}\right)\geq T\\ 0 & \mathrm{otherwise}. \end{array}\right.$$
- **Step 1 Initialization:**
  1. k:=1.
  2. Initialize the sample weights $w_{1,i}=\frac{1}{2t}$ and $\frac{1}{2s}$ for positive and negative samples, respectively.
  3. Initialize the feature set $F_{1}=\emptyset$, and voter set $U_{1}=\emptyset$.
- **Step 2 Strong Classifier Building:**
  1. Normalize sample weights so that their sum equals 1:
    (25) $$\begin{array}{c} {\tilde{w}}_{k,i}\leftarrow \frac{w_{k,i}}{\sum_{j=1}^{r}w_{k,j}}. \end{array}$$
  2. Obtain the weak classifier with feature *v* by optimization. The feature *v* has the minimal error $e_{v}$ in the mixed feature pool:
    (26) $$\begin{array}{c} \left[\begin{array}{cc} \left(v,d_{v},T_{v}\right)=\underset{l\in MFP}{\arg\min}\;e_{l}, & \;\mathrm{if}\;v\;\mathrm{is}\;a\;\mathrm{number}-\mathrm{type}\;\mathrm{feature},\\ \left(v,LUT_{v}\right)=\underset{l\in MFP}{\arg\min}\;e_{l}, & \;\mathrm{if}\;v\;\mathrm{is}\;a\;\mathrm{category}-\mathrm{type}\;\mathrm{feature}. \end{array}\right. \end{array}$$
  3. Determine the weight of the weak classifier with feature *v* using
    (27) $$\begin{array}{c} \alpha_{v}=ln\left(\frac{1-e_{v}}{e_{v}}\right). \end{array}$$
  4. $F_{k+1}=F_{k}\cup \left\{v\right\}$, add the voter to the voter set $U_{k}$:
    (28) $$\begin{array}{c} \left[\begin{array}{cc} U_{k+1}=U_{k}\cup \left(v,d_{v},T_{v},\alpha_{v}\right), & \;\mathrm{if}\;v\;\mathrm{is}\;a\;\mathrm{number}-\mathrm{type}\;\mathrm{feature},\\ U_{k+1}=U_{k}\cup \left(v,LUT_{v},\alpha_{v}\right), & \;\mathrm{if}\;v\;\mathrm{is}\;a\;\mathrm{category}-\mathrm{type}\;\mathrm{feature}. \end{array}\right. \end{array}$$
  5. Update the weight of all training samples for current strong classifier: $w_{k+1,i}\leftarrow {\tilde{w}}_{k,i}\beta^{1-\lambda }$, where λ=0 if the sample $x_{i}$ is correctly classified by the classifier with feature *v*, otherwise λ=1, $\beta =\frac{e_{v}}{1-e_{v}}$.
  6. k←k+1.
- **Step 3 Stop Condition Checking:**
  1. Check currently built strong classifier to decide whether it is finished or not. The voting result of current strong classifier built from $U_{k}$ on sample $x_{i}$ is calculated by $\sum_{f\in F_{k}}\alpha_{f}h_{f}\left(x_{i}\right)$. Sort the voting results of all training samples from small to large, and find the minimum value *T* where the detection rate satisfies DR.
  2. Use the threshold *T* to check the false alarm rate of all the training samples. If it is larger than FAR, go to step 2 to continue adding a voter to the strong classifier, otherwise, *T* is the threshold for the strong classifier, $U=U_{k}$, and building of the current strong classifier is finished.

## 5. Experiment by Hold-Out Validation

### 5.1. Dataset

There are very few available thermal image datasets for facial analysis. Some commonly used ones (e.g., UCHThermalFace [25], NVIE [26]) only contain gray level images. These images are converted from the raw data captured by thermal cameras. They lose the temperature information. Since our new feature types utilize the temperature information, we have to capture the images for our experiment.

For image capturing, we used a PI-450 thermal camera manufactured by Optris with a 62${}^{\circ }$ FOV lens and 382 × 288 pixels resolution. The camera was mounted on a tripod at a height of 1.6 m. We set the camera to raw image mode, which enables thermal images to be recorded over the temperature range from −20 ${}^{\circ }$C to 100 ${}^{\circ }$C.

**Figure 4:**
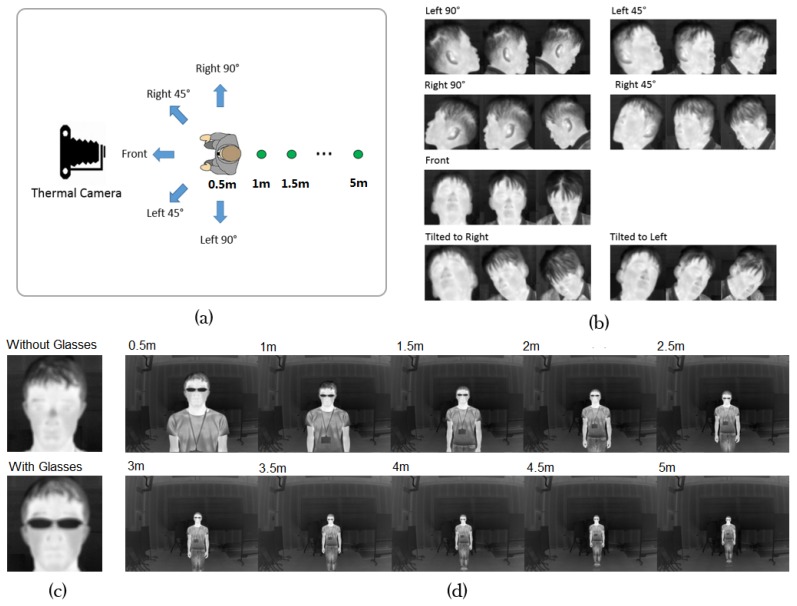
Settings and variations of our dataset. (**a**) Set-up for capturing the dataset. (**b**) Facial patches of the 21 different poses of one participant without glasses at 1 m. (**c**) Facial patches showing poses of one participant with and without glasses. (**d**) Sample images of the same participant as (**c**) with glasses standing at various distances from the camera.

We introduced three variables of participants to our dataset: distance, appearance and pose into our dataset. The first variable was distance from the camera to each participant. Ten different distances from 0.5 m to 5 m in steps of 0.5 m were set, as indicated by the green dots in Figure 4a. The second variable was appearance, which means whether glasses were worn or not. This gives two participant appearances. We show the two appearances in Figure 4c. The third variable was pose, which indicates the head posture of the participant. For each appearance and distance, we obtained 15 poses by asking the participant to face five directions (shown by blue arrows in Figure 4a) while looking up, forward, and down. Furthermore, we captured extra 6 poses of front face with head tilted on left and right sides while looking up, forward, and down. Thus we had 15+6=21 poses. We captured the extra 6 poses because statistically, there are more front-facing than side-facing images in real imaging settings. In establishing the image dataset, we employed twenty participants comprising fourteen males and six females, and obtained: 20 participants × 10 distances × 2 appearances × 21 poses = 8400 images.

After obtaining the images, we manually marked facial patches, each is defined by the square of side length equal to the height from the chin to the top of head. Figure 4b illustrates facial patches of one participant in all poses without glasses at 1 m distance.

### 5.2. Experiment Settings

In the experiment, we employed hold-out validation, which divides the whole dataset randomly into two halves with the same number of images. We used half of the images as training data and the other half of the images as testing data. We repeated this process for five times. To make the samples in the two halves similar, we adopted stratified sampling by considering the three variables in the dataset and also the gender.

In the training phase, we used the facial patches previously marked as positive samples. We used all non-facial patches in the captured images as negative samples. We set the minimum detection rate (DR) to 0.995 and the maximum false alarm rate (FAR) to 0.5 for strong classifiers. We normalized all sample sizes to 24 × 24. These parameter values are default settings in OpenCV 2.4.9.

In the testing phase, we used the sliding-window-based approach. To handle faces in images with differing scales, we built an image pyramid with multiple layers. We set the scaling factor between pyramid layers to 1.1. Furthermore, we set the minimum face size to 24 × 24, and maximum face size to 150 × 150. The reason is the statistical analysis indicated that faces in our dataset were within this range. To determine whether the detected bounding box was correct or false, the judging criteria we used was the Jaccard Index [27]:

(29) $$Jaccard\;Index\left(B_{1},B_{2}\right)=\frac{Area\left(B_{1}\cap B_{2}\right)}{Area\left(B_{1}\cup B_{2}\right)},$$

where the $B_{1}$ represents the marked bounding box of the ground truth facial region, and $B_{2}$ represents the detected bounding box. We judged $B_{2}$ as correct detection when $Jaccard\;Index\left(B_{1},B_{2}\right)$ was larger than 0.5. After face detection was completed for all layers, the results of all the layers were fused into the original scale by grouping the bounding boxes with the same facial region.

### 5.3. Training Results and Discussion

In this section, we discuss the trained cascade classifiers in three aspects. First aspect is the margin distributions of the Multi-Block LTP and AMB-LTP features in cascade classifiers. Second aspect is the reference temperature distributions of AMB-LBP and AMB-LTP features in the cascade classifiers. Third aspect is the composition of features in each strong classifier of our proposed cascade classifiers with different feature types. The first and second aspects provide insight in our new feature types. The third aspect provides insight in our cascade classifiers with multiple feature types.

**Figure 5:**
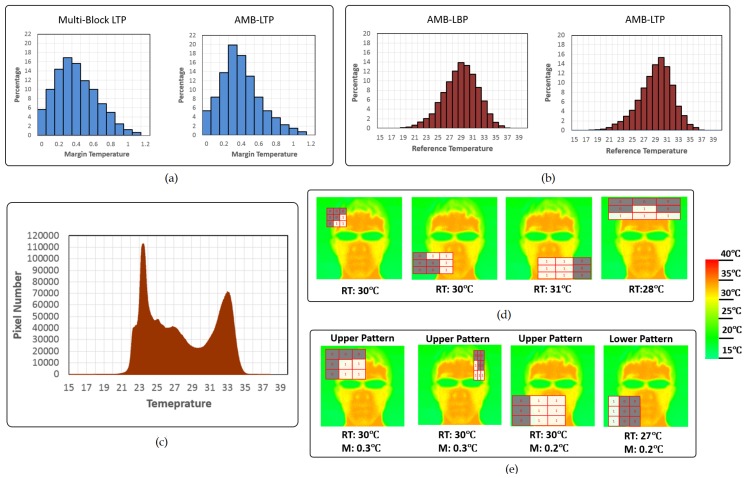
(**a**) The margin distribution of Multi-Block LTP and that of AMB-LTP by averaging those in the five times of hold-out validation. (**b**) The reference temperature distribution of the AMB-LBP and AMB-LTP by averaging those in the five times of hold-out validation. (**c**) The temperature distribution of all the pixels in the facial patches from our dataset. (**d**) Visualization of some typical AMB-LBP features from the first few strong classifiers. (**e**) Visualization of some typical AMB-LTP features from the first few strong classifiers. In (**d**,**e**) we use RT and M as the abbreviations for reference temperature and margin. The white and gray blocks in the feature represent the codes of 1 and 0, respectively.

First, we show margin distributions of the Multi-Block LTP and AMB-LTP features in cascade classifiers in Figure 5a. These cascade classifiers were trained using a feature pool only contained Multi-Block LTP features and that only contained AMB-LTP features. The distribution was obtained by averaging those in the five times of hold-out validation. We can see the margin on average is around 0.3 ${}^{\circ }$C for both Multi-Block LTP and AMB-LTP.

Second, we show the distributions of the reference temperature in Figure 5b of AMB-LBP and AMB-LTP in cascade classifiers. These cascade classifiers were trained using feature pool only contained AMB-LBP features and that only contained AMB-LTP features. The distribution was obtained by averaging those in the five times of hold-out validation. We can see that the mean reference temperatures for both feature types are around 30 ${}^{\circ }$C. To understand the meaning of this range of reference temperature, we show the temperature distribution of all the pixels in all the facial patches from our dataset in Figure 5c. From Figure 5c we can see, there are two peaks. The higher and lower peaks are from the facial skin and hair/glasses/background regions, respectively. 30 ${}^{\circ }$C is the temperature which can separate the two peaks completely. This means most features work by discriminating the facial skin and hair/glasses/background regions. We can clearly see this by the visualization of some typical AMB-LBP and AMB-LTP features from the first few strong classifiers in Figure 5d,e.

**Figure 6:**
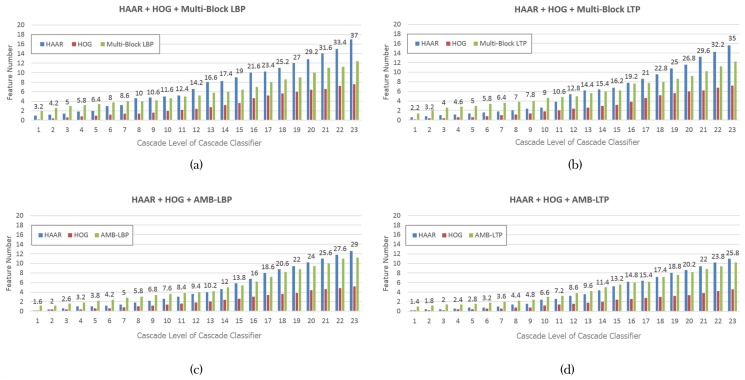
The composition of strong classifiers by average those in the five times of hold-out validation. The numbers on the bins represent the total number of features in the corresponding strong classifier. (**a**) Composition of each strong classifier trained by using feature pool {Haar-like Features, HOG Features, Multi-Block LBP Features}. (**b**) Composition of each strong classifier trained by using feature pool {Haar-like Features, HOG Features, Multi-Block LTP Features}. (**c**) Composition of each strong classifier trained by using feature pool {Haar-like Features, HOG Features, AMB-LBP Features}. (**d**) Composition of each strong classifier trained by using feature pool {Haar-like Features, HOG Features, AMB-LTP Features}.

Third, we show the composition of each strong classifier in cascade classifiers in Figure 6. These cascade classifiers were trained using our mixed feature pools. They contain Haar-like, HOG and one feature type from the set {Multi-Block LBP, Multi-Block LTP, AMB-LBP, AMB-LTP}. The composition was obtained by averaging those in the five times of hold-out validation. We can see the total number of features in strong classifiers which trained by different mixed feature pools are different. We compare the strong classifiers at the same position in cascade classifiers. The strong classifier trained by mixed feature pool: {Haar-like Features, HOG Features, Multi-Block LBP Features} uses the most number of features. The second one is the strong classifier trained by mixed feature pool {Haar-like Features, HOG Features, Multi-Block LTP Features}. The third one is the strong classifier trained by mixed feature pool {Haar-like Features, HOG Features, AMB-LBP Features}. Strong classifier used the least number of features is that trained by mixed feature pool {Haar-like Features, HOG Features, AMB-LTP Features}. This phenomenon can be explained by the fact that using features with stronger description power will result in a strong classifier with less number of features. In another words, using fewer features means each feature is more effective.

### 5.4. Testing Results and Discussion

#### 5.4.1. Discussion Based on Recall and Precision

**Figure 7:**
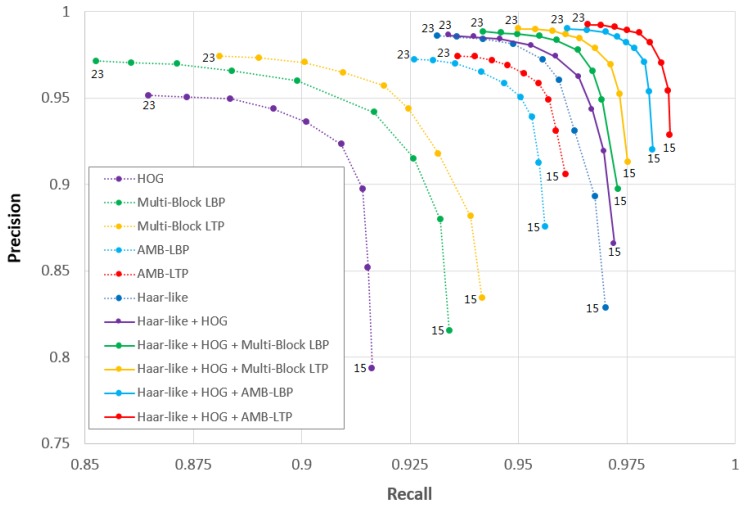
Combined results of hold-out validation experiments. The dot curves represent the performance of cascade classifiers trained by using feature pools containing single feature types, and solid curves represent those by using feature pools containing multiple feature types.

We used both recall and precision for evaluation. Recall was defined by the ratio of the number of correctly detected faces divided by the number of all faces in the testing set. Precision was defined by the ratio of the number of correctly detected faces divided by all the detected bounding boxes in the testing set. We calculated the recall and the precision of cascade classifiers with different numbers of strong classifiers from 15 to 23. These cascade classifiers were trained by using different feature pools containing either single or multiple feature types. We drew the results as curves in the coordinate of recall and precision. In this way we obtained five sets of curves for the five times of hold-out validation. We combined the these sets of curves in Figure 7. Each curve represents the performance of cascade classifiers trained by using one feature pool. Each point on the curve indicates the average in recall and precision for a specific number of strong classifiers.

Comparing the performance of different cascade classifiers trained by different feature pools is equal to comparing those feature pools. To compare different feature pools, we had a rule. We deemed the feature pool used in curve C2 is better than that used in C1, if for any point on one curve C1, we can always find a point on curve C2 with a higher recall and precision. From the curves, we can see the following aspects.

First, there is a trade-off between recall and precision from all curves. If we increase the number of strong classifiers, precision improves but recall declines. The reason is when using more strong classifiers, the cascade classifier became stricter. As a result, more facial patches were screened, which lowered the recall. Meanwhile, even more non-facial patches were screened, so the precision got improved. When using less strong classifiers, the situation was opposite.

Second, feature pools containing Multi-Block LTP and AMB-LTP are better than those containing Multi-Block LBP and AMB-LBP, respectively. This means considering a margin improved the performance. It can be explained in the same way as asserted by Tan and Triggs [15] or Jia et al. [21], that a margin brings about robustness for camera noise.

**Figure 8:**
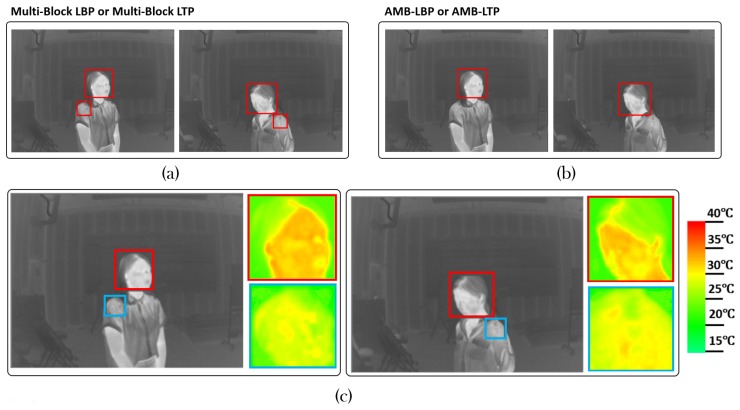
(**a**) The detection results by using cascade classifier consisted of Multi-Block LBP or Multi-Block LTP features. We can see there are non-facial patches which are hard to discriminate from facial patches. (**b**) The detection results by using cascade classifier consisted of AMB-LBP or AMB-LTP features and the same images in (**a**). We can see facial patches are correctly detected. (**c**) Temperature maps of bounding boxes from the same images in (**a**,**b**). Patches in the blue boxes have different temperature ranges with those in the red boxes. As a result, feature types considering absolute temperature can better discriminate them.

Third, feature pools containing AMB-LBP and AMB-LTP are better than those containing Multi-Block LBP and Multi-Block LTP, respectively. This means considering the absolute facial temperature improved the performance. We found some non-facial patches which were hard to discriminate from facial patches by cascade classifiers consisted of Multi-Block LBP or Multi-Block LTP features. We show them in Figure 8a, these images contain false alarm bounding boxes. However, when using cascade classifiers consisted of AMB-LBP or AMB-LTP features, they can be correctly discriminated. We show them in Figure 8b, where we can see there is no false alarm bounding box. Because these non-facial and facial patches have different temperature ranges, feature types considering absolute temperature can better discriminate them. We can clearly see this in Figure 8c, where we show the temperature maps of bounding boxes from the same images in Figure 8a,b.

Forth, we can see, the performance of the feature pools containing multiple feature types is better than those containing single feature types. This means using multiple feature types brings about stronger description power. Our feature pools containing Haar-like, HOG and one feature type from the set {Multi-Block LBP, Multi-Block LTP, AMB-LBP, AMB-LTP} are better than that containing Haar-like and HOG by Xia et al. [13]. This means mixing one more category-type feature is beneficial, because more feature types can bring about more description power.

Finally, in all the feature pools, {Haar-like Features, HOG Features, AMB-LTP Features} gives the best performance. So we see by employing all of our proposed approaches, we got the best performance.

#### 5.4.2. Discussion Based on Accumulated Rejection Rate

**Figure 9:**
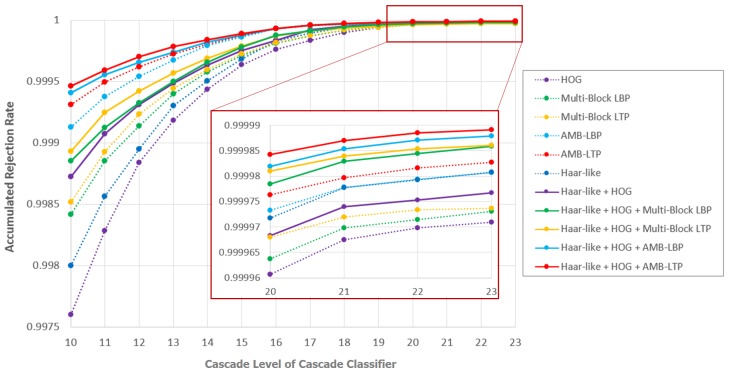
Combined results of accumulated rejection rate of cascade classifiers. The dot curves represent the accumulated rejection rate of cascade classifiers trained by using feature pools containing single feature types, and solid curves represent those by using feature pools containing multiple feature types.

By recall and precision in previous subsection, we evaluated the final detection performance of cascade classifiers trained with different feature pools. In order to understand the rejection ability of those cascade classifiers, we further evaluated the accumulation rejection rate. The accumulated rejection rate is defined as follows:

(30) $$\Gamma_{\tau ,t}=\frac{N^{p}-N_{\tau ,t}^{p}}{N^{p}},$$

where $\Gamma_{\tau ,t}$ is the accumulated rejection rate of the cascade classifier trained by feature pool τ with *t* strong classifiers. $N^{p}$ is the total number of non-facial patches cropped from all images in the testing data in all layers of image pyramids. $N_{\tau ,t}^{p}$ is the number of non-facial patches which pass the *t* strong classifiers of cascade classifier trained by feature pool τ. We measured the accumulated rejection rate of cascade classifiers trained by different feature pools in all of the five hold-out validation experiments, and averaged the results. We show the averaged accumulated rejection rate of cascade classifiers with the number of strong classifiers from 10 to 23 in Figure 9.

First, to compare the accumulated rejection rate in early phase of cascade classifiers, we check cascade classifiers with the number of strong classifiers before 15. We see cascade classifiers trained by feature pools containing AMB-LBP or AMB-LTP have high rejection ability. To be specific, cascade classifiers trained by feature pool {Haar-like Features, HOG Features, AMB-LTP Features} give the highest rejection ability, then are those trained by {Haar-like Features, HOG Features, AMB-LBP Features}. Cascade classifiers trained by feature pool {AMB-LTP Features} or {AMB-LBP Features} are weaker in rejecting non-facial patches than those trained by {Haar-like Features, HOG Features, AMB-LTP Features} or {Haar-like Features, HOG Features, AMB-LBP Features}, but better than those trained by feature pools containing feature types without considering the absolute temperature. This means considering the absolute temperature can better reject non-facial patches in early phase of cascade classifiers.

Second, to understand the convergence of accumulated rejection rate, we check the number of strong classifiers from 20 to 23. We can see even the difference is small, cascade classifiers trained by feature pools contain 3 feature types: Haar-like, HOG and one feature type from the set {Multi-Block LBP, Multi-Block LTP, AMB-LBP, AMB-LTP} converge to the highest accumulated rejection rate. Those trained by feature pool {AMB-LTP Features} or {AMB-LBP Features} still own high rejection ability. This means using a mixed feature pool containing 3 feature types can give high rejection ability for the whole cascade classifiers.

Finally, it is interesting to find that in Figure 7, Haar-like has the best overall performance evaluated by recall and precision using single feature type. However, from Figure 9 we can see its accumulated rejection rate is not the best one using single feature type. To give an intuitionistic explanation to this phenomenon, we can further derive Equation (30) to bridge the evaluation metric of recall and precision used in Figure 7 and the accumulated rejection rate used in Figure 9 as:

(31) $$\begin{array}{cc} \Gamma_{\tau ,t} & =\frac{N^{p}-{\hat{N}}_{\tau ,t}^{fp}\cdot \frac{N_{\tau ,t}^{p}}{{\hat{N}}_{\tau ,t}^{fp}}}{N^{p}} \end{array}$$

(32) $$\begin{array}{c} =\frac{N^{p}-\left[\left(1/P_{\tau ,t}-1\right)\cdot R_{\tau ,t}\cdot {\hat{N}}^{f}\right]\cdot \frac{N_{\tau ,t}^{p}}{{\hat{N}}_{\tau ,t}^{fp}}}{N^{p}} \end{array}$$

(33) $$\begin{array}{c} =\frac{N^{p}-\left[\left(1/P_{\tau ,t}-1\right)\cdot R_{\tau ,t}\cdot {\hat{N}}^{f}\right]\cdot \eta_{\tau ,t}}{N^{p}}, \end{array}$$

where ${\hat{N}}_{\tau ,t}^{fp}$ represents the number of final grouped false alarm bounding boxes in the testing dataset using the cascade classifier trained by feature pool τ with *t* strong classifiers. ${\hat{N}}^{f}$ is the total number of faces in the testing dataset. Since we used 4200 images with each image containing one face for testing, here this number is 4200. $P_{\tau ,t}$ and $R_{\tau ,t}$ are the recall and precision we used in Figure 7 with *t* strong classifiers trained by feature pool τ. $\eta_{\tau ,t}$ is highly depending on τ and *t*. We took the cascade classifier of Haar-like with 15 strong classifiers in Figure 7 as an example. We observed that at this point, recall is relatively high but precision is relatively low. From Equation (31), when recall is high or precision is low, accumulated rejection rate tends to become lower.

#### 5.4.3. Detection Time Evaluation

Even the detection time of the cascade classifiers is highly related to the implementation method, it is still worth to give comparison results for reference based on our programming. Basically, when using a cascade classifier to detect faces, a user inputs a whole image, and the output is the original image with bounding boxes. For the detection of single image, the time for the whole process using a cascade classifier with *t* strong classifiers trained by feature pool τ can be modelled as:

(34) $$T_{\tau ,t}^{total}=T_{\tau ,t}^{serv}+T_{\tau ,t}^{pass}+T_{\tau ,t}^{pos},$$

where $T_{\tau ,t}^{serv}$ is the time needed by preprocessing and system servicing. Preprocessing includes the initialization of cascade classifier, multi-scale pyramid building, patches cutting by the sliding window, etc. System servicing includes the memory allocation etc. $T_{\tau ,t}^{pos}$ is the post processing time needed after the patches in the image pyramid of the image pass the cascade classifier. Post processing includes the grouping of detected patches into bounding boxes, memory recovery, etc. $T_{\tau ,t}^{pass}$ is the time of the patches in the pyramid passing the cascade classifier with *t* strong classifiers. Because in the image pyramid, generally most patches cropped are non-facial patches, while only countable number of patches are facial-patches. If we omit the facial-patches and assume all the patches are non-facial patches, theoretically, we can model this time as:

(35) $$T_{\tau ,t}^{pass}≐\frac{{\dot{N}}_{img}^{p}}{Tr}\sum_{i=1}^{t}\left(1-\Gamma_{\tau ,(i-1)}\right)\cdot {\dot{N}}_{\tau ,i}^{fe}\cdot {\dot{t}}_{\tau ,i}^{fe},$$

where ${\dot{N}}_{img}^{p}$ is the totally number of patches cropped from all layers of the multi-scale pyramid of the image img, this number depends on the the size of img and also the situation of pyramid construction and the step length of the moving sliding window. Tr is the number of threads used in calculation. $\Gamma_{\tau ,i}$ is the accumulated rejection rate using *i* strong classifiers trained by feature pool τ. We define $\Gamma_{\tau ,0}=0$, since all the patches must pass the first strong classifier at least. ${\dot{N}}_{\tau ,i}^{fe}$ is the number of features in the strong classifier with index *i*. ${\dot{t}}_{\tau ,i}^{fe}$ is the average calculation time needed by single feature in the strong classifier with index *i*. For τ containing single feature type, ${\dot{t}}_{\tau ,i}^{fe}$ is relatively constant. For τ containing multiple feature types, ${\dot{t}}_{\tau ,i}^{fe}$ is the average calculation time for all features with different types. It is not as constant as the τ with single type, since the composition of feature types also differs for strong classifiers.

**Figure 10:**
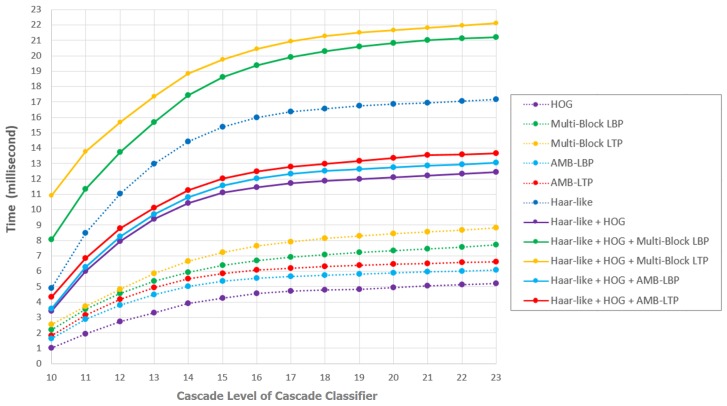
The average calculation time of patches from multi-scale pyramid of one image passing cascade classifiers with different number of strong classifiers.

We evaluated the time $T_{\tau ,t}^{pass}$ for cascade classifiers trained by all the τ we have. For measuring $T_{\tau ,t}^{pass}$, in the program, we decoupled $T_{\tau ,t}^{pass}$ and $T_{\tau ,t}^{serv}$ at the image patch calculation level. Inside the patch calculation, for cascade classifiers with multiple feature types, different functions for calculation different feature types must be called. Since calling functions also needs system time, which means cascade classifiers with multiple feature types have larger basis time than those with single feature type. Considering this fact, in later discussion, we will compare cascade classifiers with single and multiple feature types separately. We measured the $T_{\tau ,t}^{pass}$ for all 4200 images in the testing data in one of the five hold-out validation using different cascade classifiers trained with different feature pools, and repeated the measurement for five times for the five times of hold-out validation. We averaged the time of all the 4200 × 5 = 21,000 images. In this measurement, we used a PC with i7-4510 CPU and 8G memory, we used 4 threads in calculation. We show the results in Figure 10 for cascade classifiers with the number of strong classifiers from 10 to 23.

First, we consider the cascade classifiers with single feature type. From Figure 10 we can see, comparing the time used by cascade classifiers with same number of strong classifiers from 10 to 23, cascade classifiers with Haar-like features are the most time consuming ones. The reason mainly comes from the bad rejection ability of Haar-like features. The fastest cascade classifiers are those with HOG features. Even HOG also has bad rejection ability, the calculation of each single HOG feature is much faster, which leads to fast detection speed. Cascade classifiers containing Multi-Block LBP are slower than those containing AMB-LBP or AMB-LTP, but faster than those containing Multi-Block LTP. This is because AMB-LBP and AMB-LTP have stronger rejection ability, thus they achieve shorter detection time than Multi-Block LBP. Furthermore, we see even Multi-Block LTP has a little better rejection ability than Multi-Block LBP, but Multi-Block LTP is slower than Multi-Block LBP. The reason is for single feature calculation, Multi-Block LTP feature is more time consuming than Multi-Block LBP feature, since Multi-Block LTP feature is more complex. For AMB-LBP and AMB-LTP, the situation is similar as that of Multi-Block LBP and Multi-Block LTP. Overall, we see AMB-LBP and AMB-LTP which considering absolute temperature consume shorter time than Multi-Block LBP and Multi-Block LTP. The reason is considering absolute temperature can improve the rejection ability.

Second, we consider the cascade classifiers with multiple feature types. Comparing the time used by cascade classifiers with same number of strong classifiers between 10 to 23, the cascade classifiers trained by feature pools of both {Haar-like Features, HOG Features, Multi-Block LBP Features} and {Haar-like Features, HOG Features, Multi-Block LTP Features} are slower than those trained by feature pools of {Haar-like Features, HOG Features, AMB-LBP Features}, {Haar-like Features, HOG Features, AMB-LTP Features} and {Haar-like Features, HOG Features}. We see the rejection ability of cascade classifiers with {Haar-like Features, HOG Features, Multi-Block LTP Features} is better than those with {Haar-like Features, HOG Features, Multi-Block LBP Features} by a small margin, but cascade classifiers with {Haar-like Features, HOG Features, Multi-Block LTP Features} are a little slower than those with {Haar-like Features, HOG Features, Multi-Block LBP Features}. This can be explain by the fact that for single feature, the calculation of Multi-Block LTP is more time consuming than that of Multi-Block LBP. For cascade classifiers with {Haar-like Features, HOG Features, AMB-LBP Features} and those with {Haar-like Features, HOG Features, AMB-LTP Features}, the situation is similar as that of cascade classifiers trained by {Haar-like Features, HOG Features, Multi-Block LBP Features} and {Haar-like Features, HOG Features, Multi-Block LTP Features}. Overall, we can see in our proposed feature pools which contain Haar-like, HOG and one feature type from the set {Multi-Block LBP, Multi-Block LTP, AMB-LBP, AMB-LTP}, mixing feature type considering absolute temperature generally consume shorter time. The reason is considering absolute temperature can improve the rejection ability for cascade classifiers with multiple feature types.

Third, we found an interesting fact when when comparing the cascade classifiers trained with feature pools containing 3 different feature types. We can see that with the same number of strong classifiers, cascade classifiers trained by {Haar-like Features, HOG Features, Multi-Block LBP Features} or {Haar-like Features, HOG Features, Multi-Block LTP Features} consume almost double time as those trained by {Haar-like Features, HOG Features, AMB-LBP Features} or {Haar-like Features, HOG Features, AMB-LTP Features}. To intuitionistic understand this, we see two facts: (1) In Figure 9, taking 10 strong classifiers as example, the accumulated rejection rate for cascade classifiers trained by {Haar-like Features, HOG Features, Multi-Block LBP Features} or {Haar-like Features, HOG Features, Multi-Block LTP Features} is around 0.999, that for cascade classifiers trained by {Haar-like Features, HOG Features, AMB-LBP Features} or {Haar-like Features, HOG Features, AMB-LTP Features} is around 0.9995. With respect to formula (35), (1–0.999) is just double of (1–0.9995), we can see this relation keeps on from the number of 10 strong classifiers to at least 17 in Figure 9. (2) From Figure 6 we can see the number of features in cascade classifiers trained by {Haar-like Features, HOG Features, Multi-Block LBP Features} or {Haar-like Features, HOG Features, Multi-Block LTP Features} are more than those trained by {Haar-like Features, HOG Features, AMB-LBP Features} or {Haar-like Features, HOG Features, AMB-LTP Features}. By considering these two facts, we can explain the fact that cascade classifiers trained by {Haar-like Features, HOG Features, Multi-Block LBP Features} or {Haar-like Features, HOG Features, Multi-Block LTP Features} are much slower than those trained by {Haar-like Features, HOG Features, AMB-LBP Features} or {Haar-like Features, HOG Features, AMB-LTP Features}.

Finally, one thing should be noticed is that in the applications employing cascade classifiers by our implementation, we found the total time $T_{\tau ,t}^{total}$ for a cascade classifiers with multiple feature types is much more than those trained by single feature type. The additional time comes from $T_{\tau ,t}^{serv}$. For cascade classifier with multiple feature types, $T_{\tau ,t}^{serv}$ is much larger than those with single feature type. The reason is that using multiple feature types, the program must prepare the data structures related to feature types for multiple times and allocate memory for different feature types, which is time consuming.

## 6. Experiment in Real Scenes

### 6.1. Capturing Environment Settings

**Figure 11:**
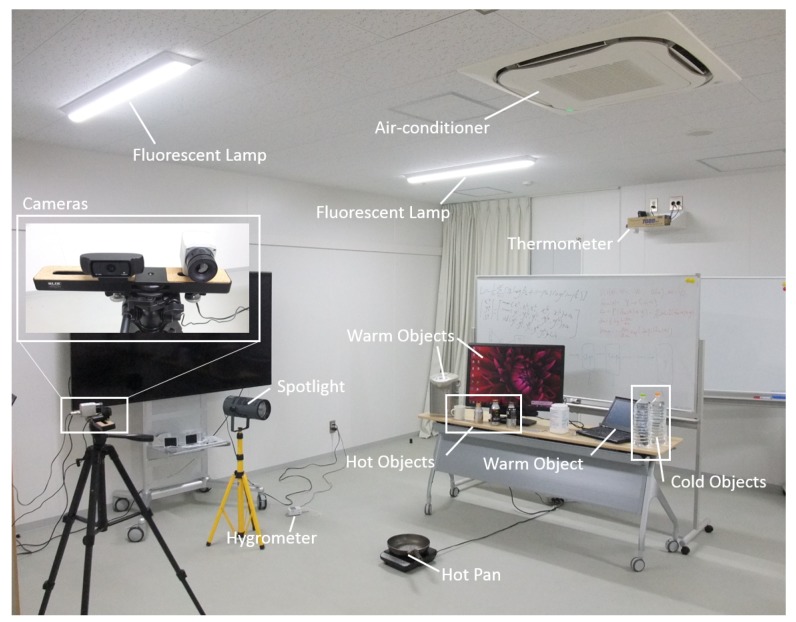
The capturing environment for our experiment.

To give evaluation of the performance of cascade classifiers in more realistic scenes for thermal images as well as RGB images, we built up an experiment environment. We show our environment in Figure 11. To make the scene more natural, we set up a background using a table and a white board. To introduce more background texture, we put some objects on the table and wrote some texts on the white board. To make the scene more challenging for face detection using thermal images, we chose the objects on the table with different temperature ranges including cold, warm and hot ones. For cold objects, we took two bottles of water from a refrigerator, they had temperature less than 10 ${}^{\circ }$C. For warm objects, we put a display, a notebook PC and a water dispenser on the table, all of them were powered to keep them warm. For hot objects, we put three aluminum bottles and a cup on the table, all of them were filled with hot water with temperature more than 35 ${}^{\circ }$C.

We controlled three environment variables: temperature, humidity, and lighting. To control temperature, we used an air-conditioner with highest temperature 30 ${}^{\circ }$C for warm mode and lowest temperature 20 ${}^{\circ }$C for cool mode (In actual testing, we found the highest and lowest temperature which the air-conditioner can reach were about 28.5 ${}^{\circ }$C and 20.5 ${}^{\circ }$C, respectively). To control humidity, we used the drying function of the air-conditioner to decrease the humidity. As for increasing the humidity, we put a boiling hot pan on the ground as steam generator. To control the lighting, we used curtains to block the windows and used fluorescent lamps and spot light. Furthermore, to accurately control the temperature and humidity, we also used a thermometer and a hygrometer to measure the temperature and humidity.

For image capturing, we used both a thermal camera and an RGB camera in order to make comparison between the two kinds of images. For thermal camera, we used a PI-450 thermal camera manufactured by Optris with the resolution of 382 × 288 pixels. We set it to raw image mode. For RGB camera, we used a C920 camera manufactured by Logicool with the resolution of 640 × 480 pixels. We adjusted to guarantee both cameras can capture the whole capturing area, which was the area between the cameras and the background. When capturing, we asked 3 participants to do some daily movements such as walking while facing the cameras in the capturing area and recorded the scene by both cameras at the same time.

### 6.2. Experiment Settings

To confirm the performance of cascade classifiers we obtained in the hold-out validation experiments in these realistic scenes, as well as make a comparison of the face detection performance under different situations using thermal and RGB images, we captured videos in 6 scenarios including a control group and 5 experiment groups. For different scenarios, we changed the environment factors such as temperature, humidity and lighting condition. The Table 1 shows the settings of the 6 scenarios.

**Figure 12:**
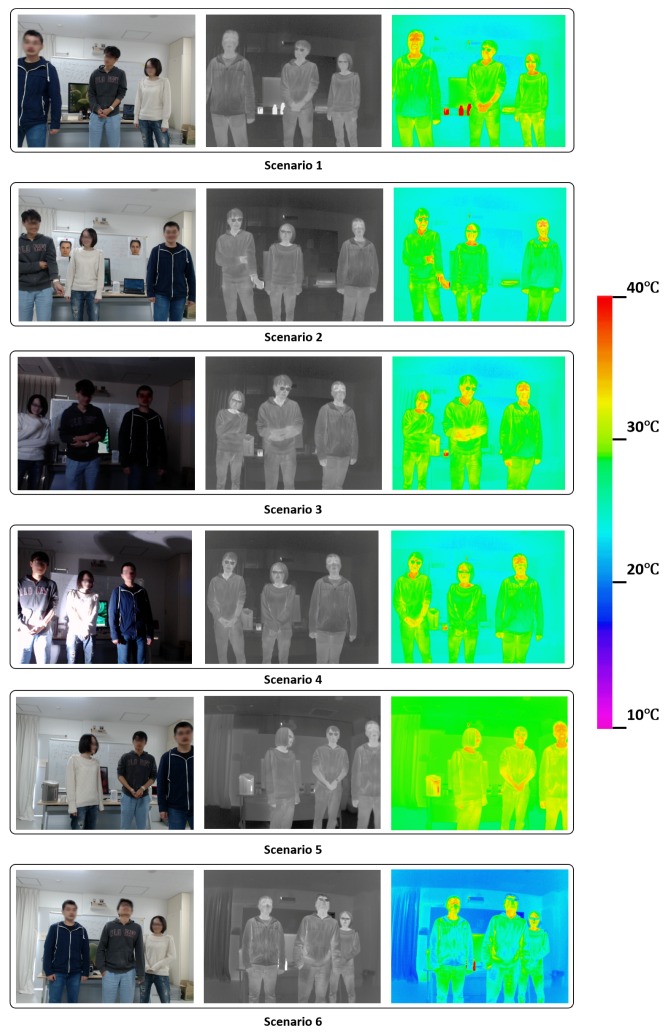
The appearances of RGB images and thermal images in the 6 scenarios. For each scenario, the left image is the RGB image, the middle one is gray level image converted from the raw image by thermal camera, the right one is the temperature map of thermal image. In this figure, for privacy protection, we slightly blurred all the facial regions of the participants in RGB images.

For the Scenario 1, we used fluorescent lamps to provide bright and uniform lighting, and set the intermediate temperature and humidity. We used the face detection results in this scenario to compare with the results from other scenarios. For the Scenario 2, we pasted 2 printed face images on the background white board, and kept other parameters same. For the Scenario 3, we closed the fluorescent lamps to make the environment uniformly dark. For the Scenario 4, we closed the fluorescent lamps and used the spot light to make the environment non-uniformly lighted. For the Scenario 5, we used fluorescent lamps to provide bright and uniform lighting, we increased the temperature. Because in natural scenes, higher circumstance temperature always corresponds to higher humidity, we also increased the humidity. For the Scenario 6, we used fluorescent lamps to provide bright and uniform lighting, we decreased the temperature. Because in natural scenes, lower circumstance temperature always corresponds to lower humidity, we also decreased the humidity. The appearances of RGB images and thermal images in all the scenarios are showed in Figure 12.

For each scenario, we captured a video about 5 min. for both thermal camera and RGB camera simultaneously. After capturing the videos in all the scenarios, we randomly selected 1000 frames of thermal images and RGB images for each scenario respectively and tested them. For thermal images we used the cascade classifiers obtained in our hold-out validation experiment. We used 20 strong classifiers for each cascade classifier. Since we have 5 cascade classifiers trained by each kind of feature pool in the 5 times of hold-out validation, we tested 5 times and averaged the results. For RGB images, we used the already trained cascade classifiers provided by OpenCV 3.3. It provides cascade classifier containing Haar-like features contributed by Howse [28] and that containing Multi-Block LBP features contributed by Puttemans et al. [29], so we used both of them.

### 6.3. Results and Discussion

For straight forward and clear comparison, we used the F-score to evaluate the performance of different cascade classifiers for thermal images and those for RGB images. The F-score is defined by

(36) $$F-score=\frac{2\cdot Recall\cdot Precision}{Recall+Precision}.$$

**Figure 13:**
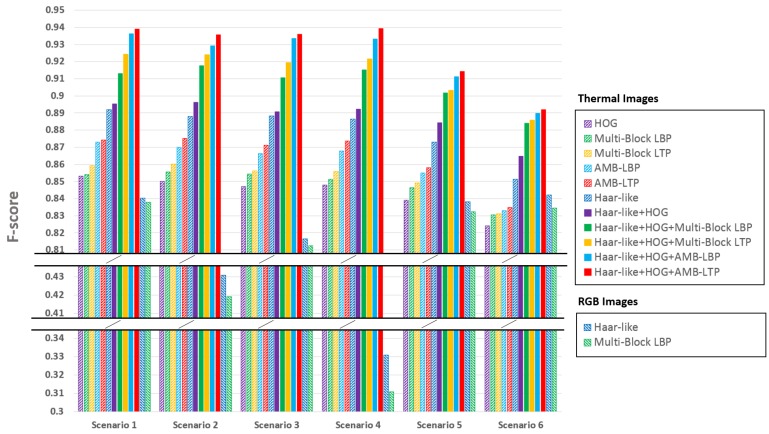
The face detection results evaluated by F-score for the 6 scenarios.

We calculated the F-scores and show the results in Figure 13 for using both thermal images and RGB images.

**Figure 14:**
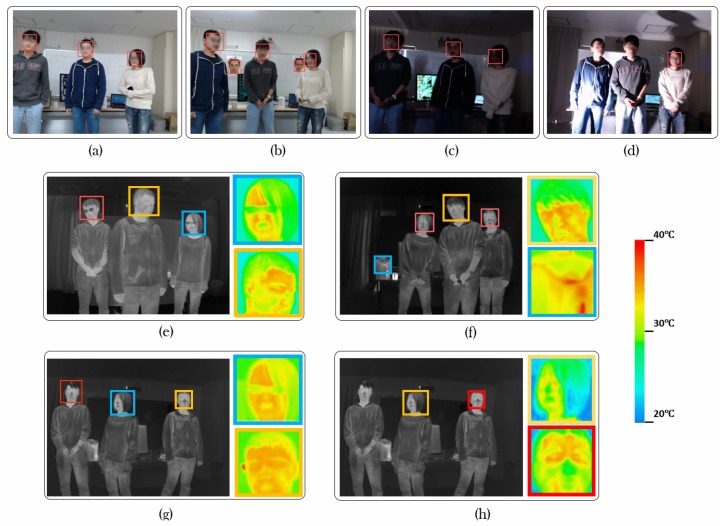
Some typical face detection results in thermal images (using Haar-like+HOG+AMB-LTP) and RGB images (using Haar-like). In this figure, for privacy protection, we slightly blurred all the facial regions of the participants in RGB images. (**a**) A typical face detection result using single RGB image from scenario 1. (**b**) A typical face detection result using single RGB image from the scenario 2. (**c**) A typical face detection result using single RGB image from the scenario 3. (**d**) A typical face detection result using single RGB image from the scenario 4. (**e**) A typical face detection result using single thermal image from the scenario 1. (**f**) A face detection result showing a false alarm by the background object. (**g**) A typical face detection result using single thermal image from the scenario 5. (**h**) A typical face detection result using single thermal image from the scenario 6. For (**e**–**h**), the bounding boxes with different color are all the detection results. We use different color to index the temperature maps.

From Figure 13 we can see, first, comparing the experiment group 1 with the control group, face detection performance using thermal images is similar. The reason is that experiment group 1 had the same temperature and humidity with those of control group. Because printed faces pasted on the white board had the background temperature, which was different from real faces, they did not affect the face detection results using thermal images. As for face detection using RGB images, we can see the printed faces lower the F-score for both the cascade classifier with Haar-like features and that with Multi-Block LBP features. The reason is the printed faces were always detected. Figure 14b shows a typical detection result using single RGB image. We can clearly see printed faces are detected.

Second, comparing the experiment group 2 with the control group, we can see face detection performance using thermal images is similar. The reason is that experiment group 2 had the same temperature and humidity with those of control group, while closing the florescent lamps did not change the environment temperature or humidity. For RGB images, the F-score for both cascade classifier using Haar-like features and that using Multi-Block LBP features drop. However, the F-score did not drop as much as the experiment group 1. The reason is that even the environment was dark, the light on the faces was quite uniform. Because uniform lighting condition does not change the relative comparison relation for Haar-like or Multi-Block LBP features, detection results did not change much. We can see a typical detection result by using single RGB image from Figure 14c, in which all the faces were correctly detected.

Third, comparing the experiment group 3 with the control group, we can see face detection performance using thermal images is similar. The reason is that experiment group 3 had the same temperature and humidity with those of control group, while spot light did not change the environment temperature or humidity. For RGB images, experiment group 3 gives the lowest F-score for both cascade classifier with Haar-like features and that with Multi-Block features. The F-scores are merely higher than 0.3. The reason is both Haar-like and Multi-Block LBP are realized by local comparison inside image patches, non-uniform lighting completely destroys the local comparative relation, thus a lot of faces cannot be detected. We can see a typical detection result by using single RGB image in Figure 14d, faces can only be detected occasionally.

Forth, comparing the experiment group 4 with the control group, we can see face detection performance for thermal images drops for all the cascade classifiers trained by different feature pools. This can be attributed to two factors: (1) The temperature distribution of human faces in warm environment changes by environment temperature and humidity. Figure 14g we give a typical detection result using single thermal image under this scenario. We cut two human faces to demonstrate the temperature distribution. Compared with those from control group in Figure 14e, we can see faces seem to be warmer. This change in facial temperature poses some difficulty for face detection using thermal images. (2) Our training dataset was captured at the similar room temperature and humidity as those of the control group. Because the background temperature and humidity were different from those of experiment group 4, the performance gets lower. However, if we also included the training data with the similar condition, we can expect a better result. For RGB images, because the lighting condition was similar to that of the control group, the results also keep similar. In this scenario, we can still see the advantage in performance by using thermal images to detect faces.

Fifth, comparing the experiment group 5 with the control group, we can see face detection performance for thermal images drops for all the cascade classifiers trained by different feature pools even more than that of the experiment group 4. We can explain this by the same two factors in comparing the experiment group 4 with the control group. In Figure 14h, we can see the face temperature distribution under this scenario. However, we can still see that the best performed cascade classifier for thermal images still owns a large margin in F-score compared with those using RGB images.

Sixth, from the experiment group 2, 3 and experiment group 4, 5, we can see lighting condition and environment temperature can affect the pixel values on facial regions for RGB and thermal images, respectively. However, they differ in extent with respect to the camera dynamic ranges. For RGB images, we checked the pixel values on the facial regions for the scenario 4 in Figure 12, we found a lot of saturated pixels. Since we used an 8-bit camera, the values of these saturated pixels are 255 in the image channels. On the other hand, we also found a lot of dark pixels on the facial regions with the pixel values close to 0. This means using an RGB camera, the changing of pixel values on the facial regions could be nearly 100% of camera dynamic range. In contrast, for thermal images, we found the highest pixel values on facial regions under scenario 5 (highest background temperature in all scenarios) are about 38 ${}^{\circ }$C (about highest body temperature), while the lowest pixel values on facial regions under scenario 6 (lowest background temperature in all scenarios) are about 21 ${}^{\circ }$C (a little higher than the background temperature). Since our thermal camera works in the range from −20 ${}^{\circ }$C to 100 ${}^{\circ }$C, [21 ${}^{\circ }$C, 38 ${}^{\circ }$C] only accounts for a small part of the whole dynamic range [−20 ${}^{\circ }$C, 100 ${}^{\circ }$C]. Taking a look at the gray level images in the middle column in Figure 12, we can see the advantage of the smaller relative change of pixel values on facial regions in camera dynamic range, that even under different environment temperature and humidity, facial regions in thermal images are visually quite similar. However, the large change of pixel values on facial regions in camera dynamic range makes the RGB images visually quite different in the first column of Figure 12. This advantage of much smaller change of pixel values on facial regions in camera dynamic range gives thermal images more room in improving face detection performance. We believe by adding more training sample variations or/and using some processing methods, the detection performance can be improved further.

Seventh, we can see from Figure 12 and Figure 14, using thermal images to detect faces can achieve better privacy protection. For RGB images, since we can easily figure out the identity of a person from facial appearance, in the demonstrated figures, we had to blur the faces to protect the privacy of the participants. In contrast, we did not need to blur facial regions in thermal images. For the applications where finding facial regions and privacy protection are both important (such as face counting in places where privacy protection is important), a thermal camera is a better choice than an RGB camera.

In conclusion, in this experiment, we showed that for face detection using RGB images, non-uniform lighting and fake faces can easily make the cascade classifiers unworkable. For face detection in thermal images, environment temperature and humidity can affect the face detection results by affecting the facial temperature distribution. However, we can see facial temperature distribution in thermal images is much more constant than facial brightness distribution in RGB images. The reason is the change of pixel values on facial regions for thermal images under different environment temperature is much smaller than that of RGB images under different lighting condition with respect to the whole camera dynamic range. Furthermore, sometimes background hot objects become false alarms for face detection using thermal images, as showed in Figure 14f, but such cases are rare compared with the difficulty caused by fake faces for RGB images. In all the experiment groups as well as the control group, we can see an advantage in face detection performance by using thermal images over that by using RGB images.

## 7. Conclusions and Future Work

In this research we employed thermal cameras for face detection. We proposed two approaches relying on local features for face detection in thermal images: (1) We created new feature types by extending Multi-Block LBP. By considering a margin and facial temperature, we improved the robustness to thermal camera noise and effectiveness for face detection. We obtained a set of feature types: {Multi-Block LBP, Multi-Block LTP, AMB-LBP, AMB-LTP}. (2) We proposed an AdaBoost-based training method to obtain cascade classifiers with multiple feature types: Haar-like, HOG and one feature type from the set {Multi-Block LBP, Multi-Block LTP, AMB-LBP, AMB-LTP} for an improved discrimination ability. We captured a dataset of 8400 images, used a hold-out validation to analyze and compare the performance of cascade classifiers trained by using single feature type and multiple feature types. The experiment results showed that our approaches improve the performance of cascade classifiers effectively, and the best result comes from the one employing all of our approaches. To test our proposed methods in more realistic scenes and make comparison with face detection using RGB images, we also did a field experiment. In the experiment, we showed the factors which affect the face detection performance for using both thermal and RGB images. We showed and discussed the advantage of face detection using thermal images.

In the future, we have two plans: (1) We intend to increase the number of samples, and add more variations to our dataset; (2) We intend to use our dataset to train CNN models and test them as another research topic.

## Figures and Tables

**Table 1:** The parameter settings and descriptions for all the scenarios we used for capturing.

	Temperature	Humidity	Lighting Condition	Other Factor	Function
Scenario 1	24 ${}^{\circ }$C	45%	Fluorescent Lighting	-	Control Group
Scenario 2	24 ${}^{\circ }$C	45%	Fluorescent Lighting	Face Images	Experiment Group 1
Scenario 3	24 ${}^{\circ }$C	45%	No Lighting	-	Experiment Group 2
Scenario 4	24 ${}^{\circ }$C	45%	Spot Lighting	-	Experiment Group 3
Scenario 5	28.5 ${}^{\circ }$C	65%	Fluorescent Lighting	-	Experiment Group 4
Scenario 6	20.5 ${}^{\circ }$C	25%	Fluorescent Lighting	-	Experiment Group 5
